# Impact of Selected Metal Oxides on the Thermodynamics of Solid Rocket Propellant Combustion

**DOI:** 10.3390/molecules31030436

**Published:** 2026-01-27

**Authors:** Kinga Janowska, Sylwia Waśkiewicz, Paweł Skóra, Lukasz Hawelek, Piotr Prasuła, Tomasz Jarosz, Agnieszka Stolarczyk

**Affiliations:** 1 Department of Physical Chemistry and Technology of Polymers, Silesian University of Technology, 44-100 Gliwice, Poland; 2 Department of Inorganic Chemistry, Analytical Chemistry and Electrochemistry, Silesian University of Technology, 44-100 Gliwice, Poland; 3 Institute of Non-Ferrous Metals, Lukasiewicz Research Network, 44-100 Gliwice, Poland; 4 Military Institute of Armament Technology, 05-220 Zielonka, Poland

**Keywords:** combustion, rocket propellant, activation energy, DSC

## Abstract

A series of catalytic oxides (Fe_2_O_3_, CuO, ZnO, and Cu_2_O) were investigated as prospective additives shaping the thermal features of a model solid rocket propellant (SRP) formulation utilising ammonium nitrate as the oxidising agent. An extensive investigation of the thermal behaviour (DSC and ignition/explosion temperature studies) of the model and catalyst-bearing SRP formulations was conducted, providing insights into both the thermodynamics and mechanism of combustion of these systems. XRD analysis of post-combustion residues was used to validate the mechanistic claims, as well as to provide information about the behaviour of copper oxides in the SRP system. In addition, the linear combustion velocity was experimentally determined, and the power output was estimated from density, linear combustion velocity and DSC data, in order to assess the potential motor performance of the tested formulations. The obtained results show that the utilisation of metal oxides significantly improves the combustion performance of ammonium nitrate-based SRP formulations relative to the unmodified ammonium nitrate-based propellants.

## 1. Introduction

Solid rocket propellants (SRPs) have found extensive application, particularly in space exploration and aeronautics [1,2,3]. Currently, ammonium perchlorate (AP) is the most common oxidising agent used in their formulations [4,5]. The use of AP in solid propellants, however, results in emissions of problematic substances (e.g., hydrogen chloride and chlorine at lower loadings) [6,7,8].

These emissions, as well as the problematic nature of perchlorate compounds, have brought about a number of research efforts dedicated to eliminating perchlorates from SRP formulations through the use of alternate oxidising agents [9,10,11]. The replacement of perchlorate in SRPs, however, is hindered by the fact that the proposed alternative oxidising agents tend to be burdened by a variety of issues, such as high toxicity and mutagenic properties (e.g., ammonium dinitramide [12]), requiring multi-stage synthesis that involves hazardous reagents and producing copious amounts of toxic/environmentally harmful waste [13], and having much higher unit costs than AP or resulting in drastically decreased performance of the SRP when compared with analogous AP-containing SRP formulations. Apart from subpar SRP performance, the listed issues cannot be resolved when maintaining the use of a particular oxidising agent, as they are intrinsic to that oxidising agent.

In light of the above, significant research efforts have been dedicated to augmenting the performance of the less problematic and inexpensive oxidising agents, such as ammonium nitrate and hydroxylammonium nitrate [14,15,16], through the use of decomposition catalysts. While the kinetic aspects of such modification have been extensively studied, relatively little attention has been dedicated to the influence of catalysts on the mechanism and thermodynamics of the combustion of SRP formulations.

Consequently, in this work, four catalytic oxides (Fe_2_O_3_, CuO, Cu_2_O, and ZnO) were selected and the effect of their inclusion in a model SRP formulation (coded as SRP-0) on both the thermodynamics of SRP combustion and the thermal properties of the modified SRP formulations was investigated.

## 2. Results and Discussion

It is known that the presence of grit in solid energetic materials influences their friction and impact sensitivity, as well as their ignition temperature. The melting point and hardness of the grit have been identified as significant factors for the increase in sensitivity and decrease in ignition temperature [17]. From a mechanistic viewpoint, the four catalytic oxides utilised in this work (Fe_2_O_3_, CuO, Cu_2_O, and ZnO) can be perceived as such grit. Consequently, the friction sensitivity and ignition/explosion temperatures of the resultant SRP formulations (Table 1) should be analysed taking into consideration the hardness and melting points of the introduced “grit” (Table 2).

The sample containing Fe_2_O_3_ was found to be the most sensitive to friction (Table 1). Materials with lower melting temperatures (e.g., CuO) are expected to have lower friction sensitivity. Additionally, low-hardness materials may be easily deformed, which prevents local accumulation of energy. Thus, softer materials are less sensitive to friction.

All samples containing metal oxides showed a lower ignition/explosion temperature than the sample containing only ammonium nitrate. The ignition/explosion of samples doped with copper oxides (CuO and Cu_2_O) were, respectively, the lowest and second lowest from among the tested samples (Table 1). As the total mass proportion of oxides in the formulations of solid rocket propellants is low, the ignition temperature changes are not particularly pronounced. Nevertheless, the decrease in ignition/explosion temperature confirms the catalytic effect of the metal oxides used on the oxidising agent decomposition and, consequently, on the entire energetic decomposition of the rocket propellant.

It is important, from the point of view of solid rocket propellants, to study their decomposition process in an oxygen-poor environment. The DTA/TG spectra presented in this publication allow the characterisation of the thermal decomposition of rocket propellants, both in air and in argon atmospheres.

**Figure 1:**
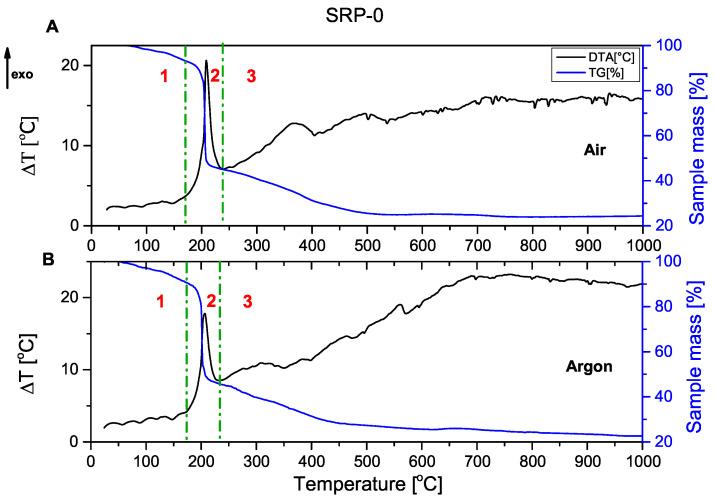
DTA/TG of SRP-0 (containing no metal oxide), recorded in air (**A**) and in argon (**B**). The dashed lines delineate the main combustion event, with individual combustion phases (1–3) being discussed below.

**Figure 2:**
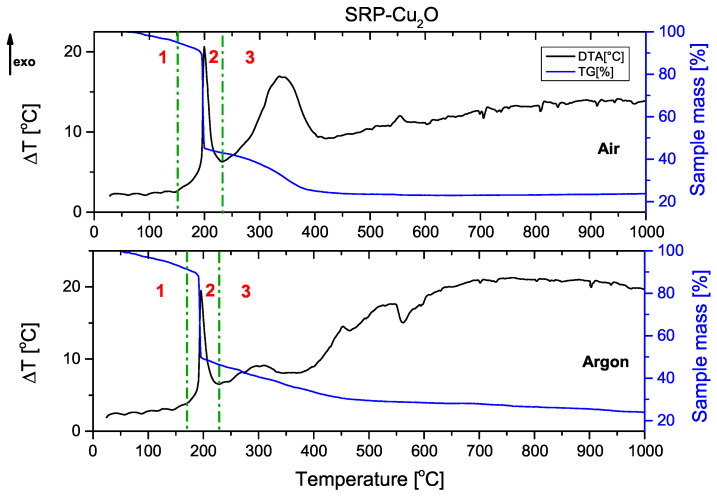
DTA/TG of the sample with copper (I) oxide (comparison of thermal decomposition occurring in air and in argon atmosphere). The dashed lines delineate the main combustion event.

The combustion process of the unmodified rocket propellant (Figure 1) and propellant containing copper(I) oxide (Figure 2) as a catalyst for oxidising agent decomposition is presented below. In each of the graphs shown, it can be seen that the decomposition of rocket propellants is not a one-step decomposition. The TG curve shows that after the initial stage of combustion (mass decrease of about 50%), propellants continue to burn until they reach a residual mass of approx. 20%. Solid residue represents the magnesium oxide formed by the oxidation of metallic fuel.

(1) $$2Mg+O_{2}\to 2MgO$$

Graphs are divided into three parts, corresponding to the three stages of combustion:

1—pre-ignition phase; 2—main combustion; and 3—afterburning.

Comparing TG and DTA curves, it can be concluded that the decomposition process occurs similarly. The DTA curve of SRP-Cu_2_O again confirmed the occurrence of secondary processes.

**Figure A4:**
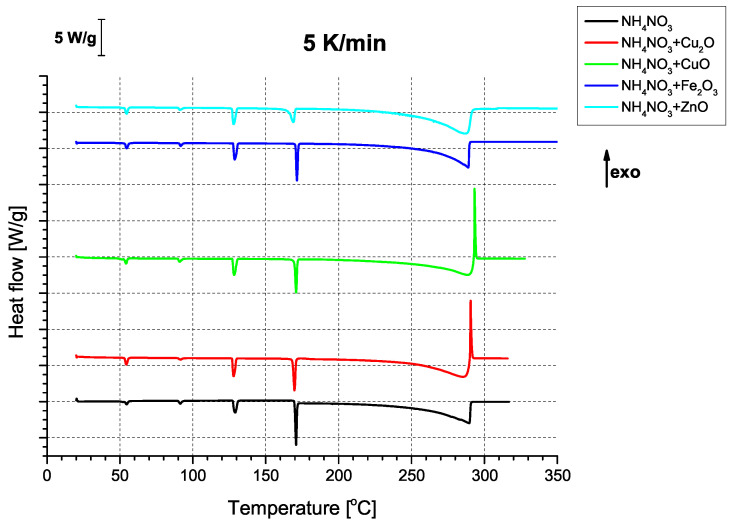
Summary thermograms of the tested samples for a heating rate of 5 K/min.

**Figure A5:**
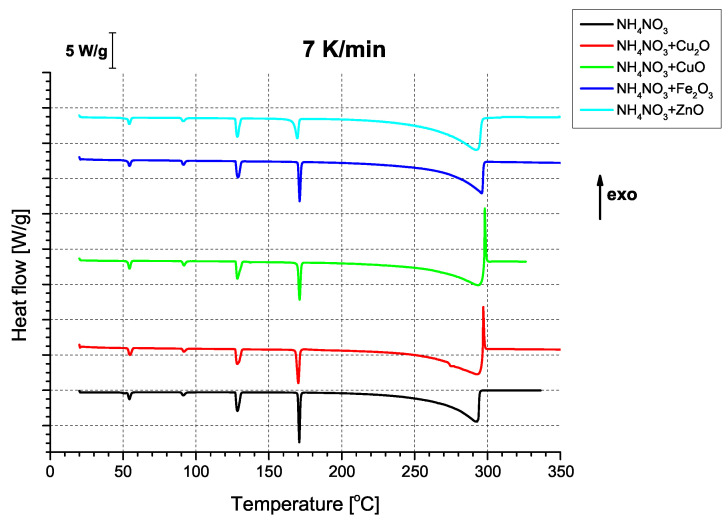
Summary thermograms of the tested samples for a heating rate of 7 K/min.

**Figure A6:**
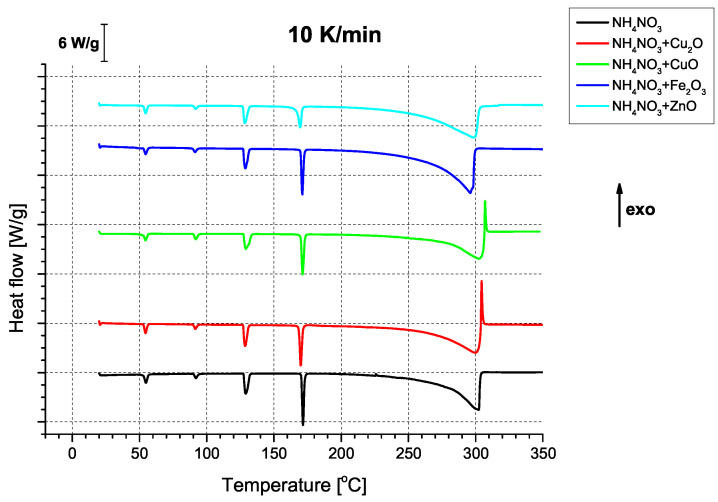
Summary thermograms of the tested samples for a heating rate of 10 K/min.

**Figure A7:**
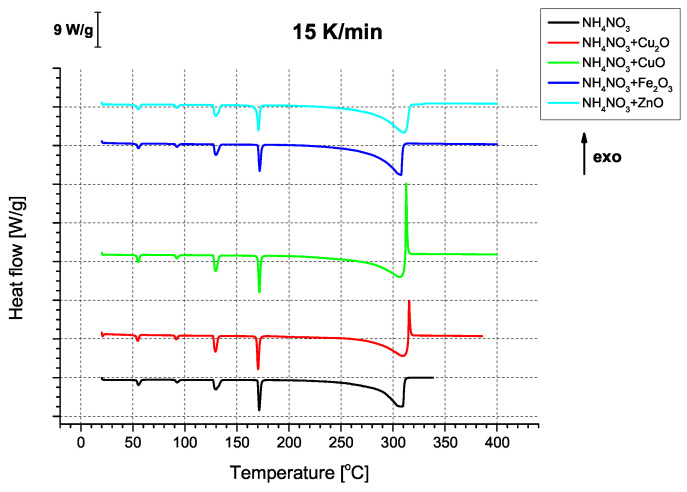
Summary thermograms of the tested samples for a heating rate of 15 K/min.

**Figure A8:**
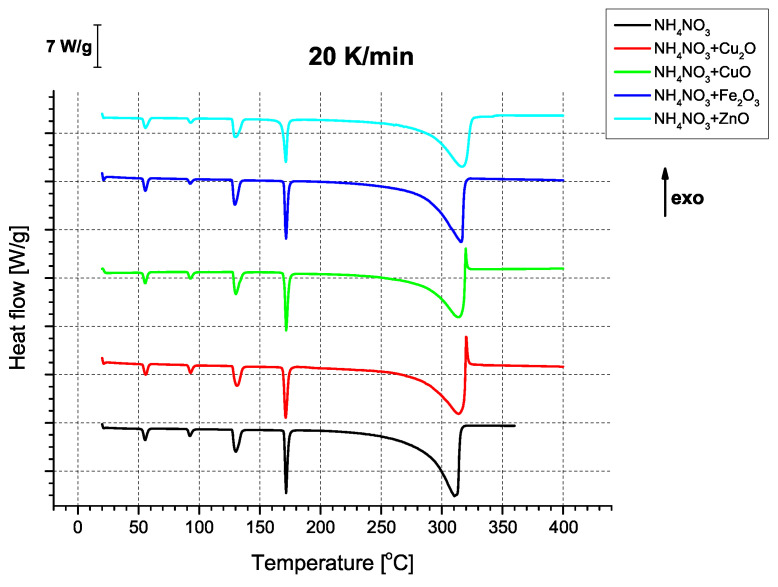
Summary thermograms of the tested samples for a heating rate of 20 K/min.

The thermograms recorded for the unmodified SRP formulation (Figure 1) and the Cu_2_O-modified SRP (Figure 2) show similar behaviour in the range of 20–200 °C. The observed endothermic peaks are associated with phase transitions of the utilised oxidising agent (these are more pronounced in Figure A4, Figure A5, Figure A6, Figure A7 and Figure A8). In the range of 200–250 °C, the intense exothermic peak originates from the oxidation of the binder and auxiliary metallic fuel in the main combustion phase. Peaks that can be observed at higher temperatures are associated with afterburning processes.

To quantify the power output of the tested formulations during combustion in a solid rocket motor, estimates were derived using the LCV values, measured density, and thermal data obtained from DSC (Table 3). Based on the known tube geometry, first the volumetric combustion rate and next the mass combustion rate were calculated. The power output was then estimated based on the heat of combustion of a unit mass of the propellant, which was obtained from DSC measurements.

Due to the determination of the combustion heat from DSC data, the obtained value is expected to be lower than the actual heat of combustion. This was reported on in paper [24] and stems from the nature of the DSC experiment as compared with typical combustion processes. Furthermore, the afterburning stage of combustion is not included in our evaluation, as it is highly dependent on actual combustion conditions and the observed secondary exothermic peaks may be specific to the conditions of the DSC experiment. Unfortunately, measurements taken using a diathermic calorimeter were found to be unreliable; hence, the DSC-derived value is provided as a less accurate estimate for the heat of combustion. Therefore, although DSC data offer valuable comparative information, they fail to represent the true enthalpy release under realistic rocket motor operating conditions, where chamber pressure, turbulent flow fields, and afterburning phenomena substantially affect energy output.

The reference sample (SRP-0) exhibited (Table 3) the lowest linear combustion velocity (1.10 mm/s) and the lowest estimated power output (36.5 W). This confirms its limited energetic performance and highlights the necessity of supplementing it with catalytic additives to improve combustion characteristics. The addition of Cu_2_O resulted in the highest linear combustion velocity (2.56 mm/s), more than twice that of SRP-0. This may be attributed to the redox properties of Cu_2_O, which can easily oxidise to CuO during thermal decomposition, facilitating multi-step electron transfer. Additionally, the mixed-valence state of copper(I) oxide may provide more efficient pathways for catalysing AN decomposition compared to the more stable Cu_2+_ in CuO [25,26].

This indicates that Cu_2_O effectively accelerates flame-front propagation, most likely due to its catalytic action on the decomposition of the oxidising agent. However, it is important to emphasise that an increased burning rate alone does not determine the energetic performance of the formulation. The final estimated power output depends simultaneously on the mass burning rate and the combustion heat obtained from DSC measurements. In this regard, Fe_2_O_3_ as well as Cu_2_O provide the most favourable combination of parameters, resulting in the highest calculated power output (107.7 W and 108.4 W respectively). These results show that Cu_2_O plays a dual role, simultaneously promoting flame propagation and improving the efficiency of energy release, making it the most effective additive among the tested additives.

The utility of CuO and ZnO is intermediate between that of Cu_2_O and Fe_2_O_3_, with LCV values of 1.41 mm/s and 1.68 mm/s, respectively. Although the power output is less than in the case of Fe_2_O_3_ and Cu_2_O, it is twice as high as that of SRP-0. This indicates that CuO and ZnO act primarily as catalysts for AN decomposition rather than as combustion promoters. As such, their inclusion in phase-stabilised AN (PSAN) [27] gives the additional utility of improving the combustion performance of any formulations containing such CuO-or ZnO-bearing PSAN.

**Figure 3:**
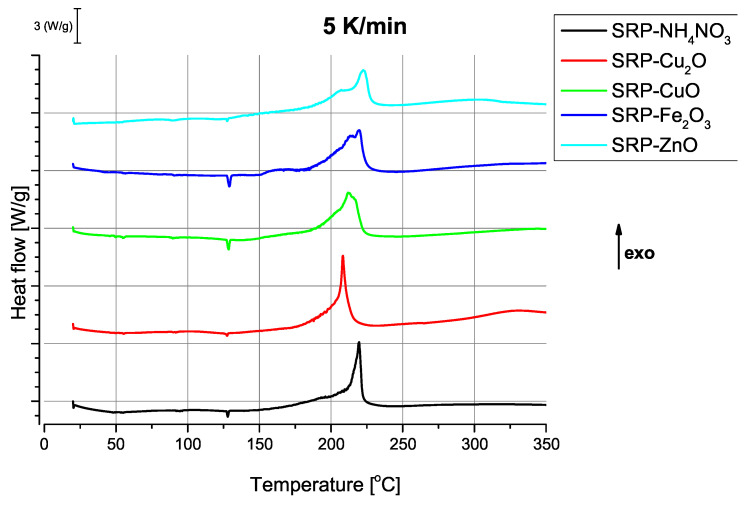
Thermograms recorded at a heating rate of 5 K/min for the studied solid rocket propellant samples.

The thermograms recorded for the solid rocket propellant samples (Figure 3) show a discernible influence of the choice of oxide on the combustion process. Although the addition of the oxide in each case results in lowering the observed ignition temperature compared to the unmodified SRP formulation; the nature of the influence of each oxide is not straightforward, as the shape and nature of the signals observed for the combustion events differ significantly.

### 2.1. Investigation of Catalytic Influence of Oxides on Ammonium Nitrate Decomposition

The investigated solid rocket propellant (SRP) samples contain multiple interacting components, resulting in the above-observed non-obvious reaction sequence. The general, well-established SRP combustion mechanism can be outlined as follows [28,29]:

- Upon ignition, endothermic decomposition of the oxidising agent takes place, resulting in the evolution of oxygen. This is frequently the limiting step of the process.
- The incipient oxygen reacts with fuels (binder and auxiliary fuels, if present) in a highly exothermic oxidation process (multiple individual and competing reactions are typically observed).
- The heat released during oxidation is partially lost with the emitted combustion products due to conductive and radiative transfer away from the reaction zone. Simultaneously, however, it is consumed to induce decomposition of the oxidising agent in adjacent layers of the SRP, sustaining and propagating the combustion front across the available SRP.

In the case of the studied SRP samples, both metallic fuels and an energetic material (nitroguanidine) have been used, ensuring that the above-mentioned reaction with the released oxygen will be extremely rapid. Consequently, decomposition of ammonium nitrate (AN) is expected to be the rate-limiting step of the process. In light of this fact, in order to elucidate the mechanism by which the oxide additives influence the combustion process, we have elected to investigate them in simplified systems, i.e., binary mixtures of AN with one of the catalytic oxide additives.

Conducting the investigation for these binary systems not only screens off processes originating from the other components of the SRP and averts the issue of cross-influences, but provides a more detailed insight into the impact of each oxide on the thermal features of AN. An important consideration at this stage is that the AN/oxide systems are composed of two separate phases, and therefore the effective contact surface between the component particles will limit the catalytic efficacy of the oxides. In general, a smaller particle size of the catalyst is typically considered desirable [30,31,32] in heterogeneous processes, yet it is not always achievable. In our case, while the four tested oxides differed in average particle size (Table 5), they were all much finer than the AN used as the oxidising agent.

**Figure 4:**
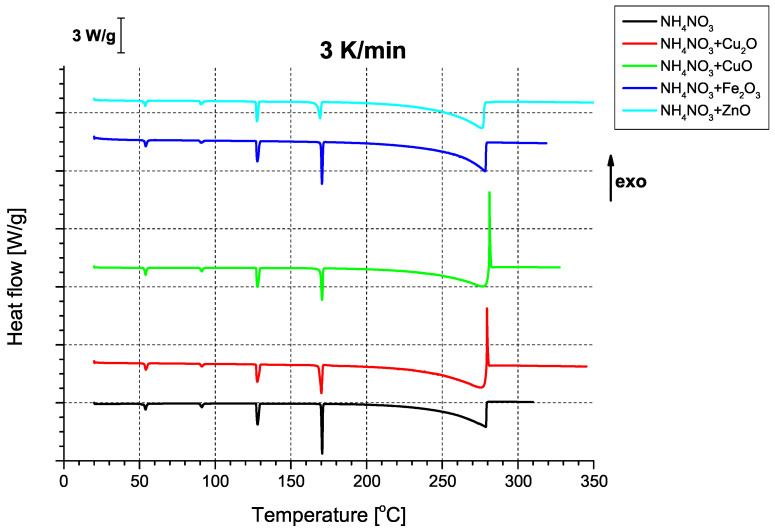
Summary thermograms of binary AN mixtures with the tested oxide additives, recorded at a heating rate of 3 K/min.

In terms of thermal properties (Figure 4), the binary AN/oxide systems all show the series of endothermic signals characteristic of the phase transitions of AN [33], regardless of the utilised heating rate (Figure A4, Figure A5, Figure A6, Figure A7 and Figure A8). Interestingly, two of the oxides, i.e., CuO and Cu_2_O, had an unexpected effect on thermally-induced AN decomposition; that is, they gave rise to a sharp exothermic peak at approx. 280 °C, occurring at the apex of AN decomposition. This is indicative of a deviation from the decomposition mechanism observed for both pure AN and binary mixtures of AN with the other two oxides (purely endothermic decomposition). Although quantitative differences between these three samples can be observed, they are better evidenced by the change in apparent activation energies (Table 4).

**Figure A9:**
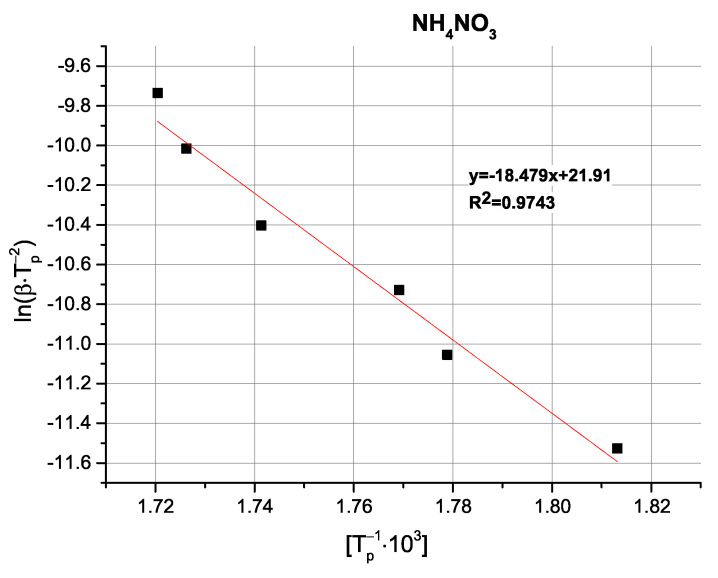
Kissinger plot of NH_4_NO_3_.

**Figure A10:**
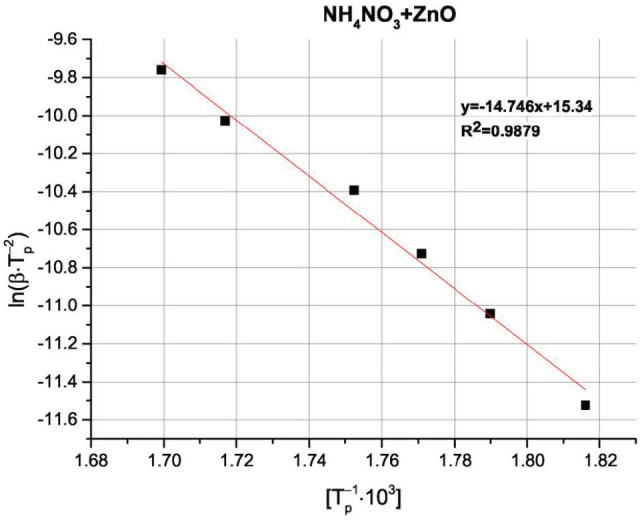
Kissinger plot of mixture of NH_4_NO_3_ and ZnO.

**Figure A11:**
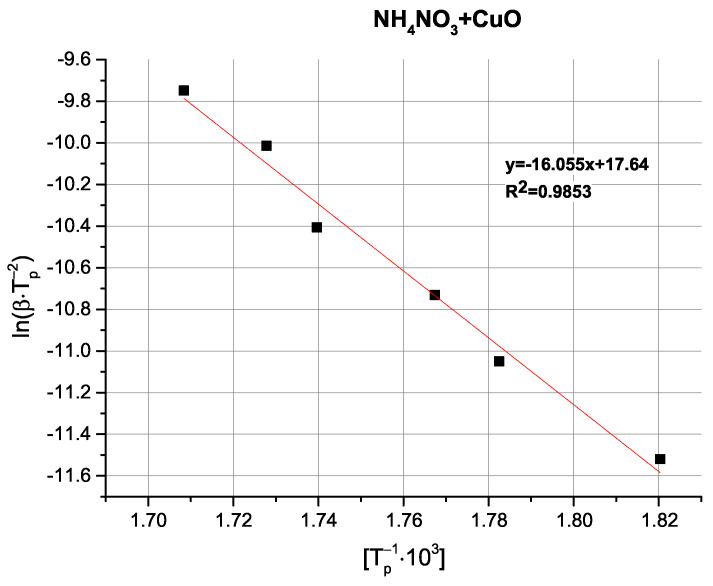
Kissinger plot of mixture of NH_4_NO_3_ and CuO.

**Figure A12:**
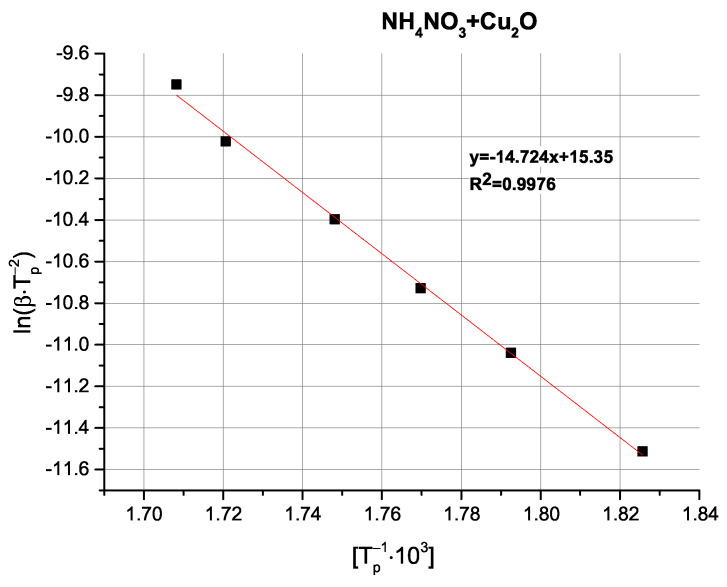
Kissinger plot of mixture of NH_4_NO_3_ and Cu_2_O.

**Figure A13:**
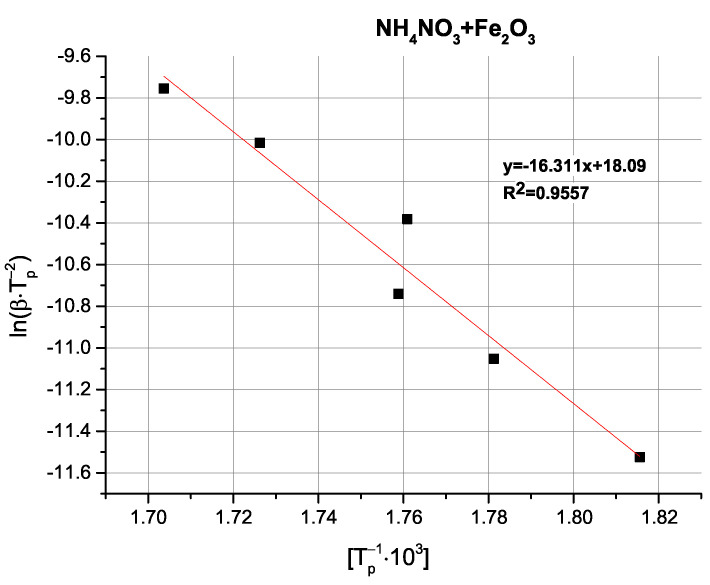
Kissinger plot of mixture of NH_4_NO_3_ and Fe_2_O_3_.

Comparison of the determined activation energy values appears to follow expectations; i.e., the highest activation energy is observed for pure AN, with the tested oxides exhibiting varying magnitudes of influence. Interestingly, the above-mentioned partially exothermic deviation from the decomposition mechanism seen for CuO and Cu_2_O is not reflected as either a marked change in activation energy or even as a non-linearity in the Kissinger plots (Figure A9, Figure A10, Figure A11, Figure A12 and Figure A13), with the ZnO-modified sample exhibiting an activation energy that is virtually as low as the activation energy determined for the Cu_2_O-modified sample. An important observation at this point is that the noticeable changes in activation energy do not directly translate to changes in the ignition/explosion temperature of the SRP (Table 1), providing justification for the study of simplified, binary systems.

### 2.2. Comparison of the Behaviour of Catalytic Metal Oxides in Binary Mixtures with AN and in SRP Formulations

**Figure 5:**
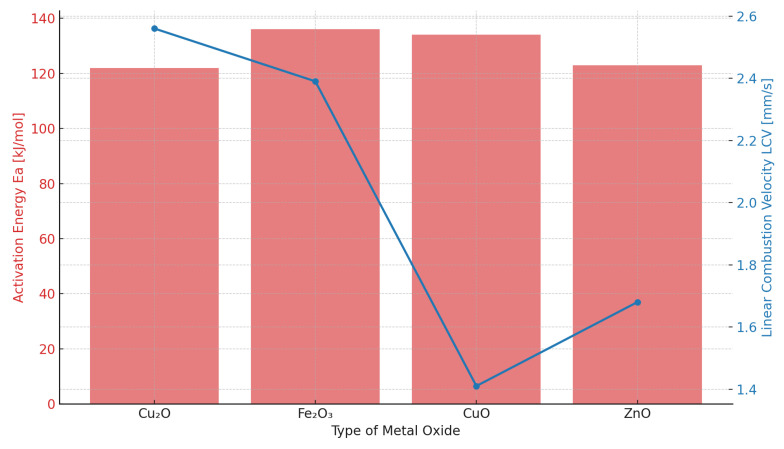
Comparison of activation energy ($E_{A}$) determined for binary systems and linear combustion velocity (LCV) determined for SRP samples.

The obtained experimental results show that the influence of metal oxides on AN thermal decomposition does not translate directly to their influence on SRP combustion, as seen by the collated comparison (Figure 5). In the simplified binary (AN/oxide) system, the lowest activation energy values were observed for AN modified with Cu_2_O and ZnO, providing evidence for their strong catalytic effect towards the AN decomposition reaction. Conversely, in the more complex SRP formulation systems, the highest linear combustion velocity (LCV) was recorded for Cu_2_O- and Fe_2_O_3_-modified samples.

The influence of Fe_2_O_3_ is more apparent during SRP combustion, where it may react with the introduced metallic fuel (Mg) in a thermite-like reaction, contributing to increasing temperature in the reaction zone, therefore increasing the propagation rate of the combustion front. In turn Cu_2_O, due to the reversible Cu^+^ ↔ Cu^2+^ redox process, can decrease the AN decomposition activation energy and increase LCV through this mechanism, which is in line with both the experimental data and the following results of identification of solid post-combustion residues.

In comparison, CuO exerts a lesser influence in both binary mixtures and in SRPs, which likely stems from its higher thermal stability and lack of ability to undergo redox transitions. In the case of ZnO, despite the significant influence on AN decomposition, a very limited effect on LCV is observed, possibly due to excess oxygen being released from the oxidising agent in the pre-ignition phase.

### 2.3. Mechanistic Implications for the Catalytic Influence of Metal Oxides

The observed changes in ignition/explosion temperatures and increased linear combustion velocity (Table 4) for the oxide-modified SRP samples, as well as the reduction in AN decomposition activation energy in binary systems, all provide ample evidence for the catalytic influence of the studied oxide additives on the thermally induced AN decomposition reaction. An important observation here is that the above parameters are influenced differently by the individual oxides; i.e., the lowest activation energy values for AN decomposition (in binary systems) are observed for Cu_2_O and ZnO, whereas the highest linear combustion velocities (for SRPs) are recorded for Fe_2_O_3_ and Cu_2_O. This is indicative of the occurrence cross-influences, such as, e.g., reductive decomposition of nitroguanidine by Fe_2_O_3_ becoming an auxiliary source of oxygen [34]. While a specific combustion mechanism would necessitate a dedicated and separate research effort, a summary of the relevant catalytic activity of each tested oxide has been collated below, allowing several implications to be drawn:

- Copper(I) oxide, Cu_2_O, readily transitions into CuO in oxidising conditions, enabling it to participate in a broad range of redox reactions and facilitating multi-step electron transfers, accelerating the decomposition of various oxidising agents. Multiple reports, e.g., [25,26], have provided evidence for the catalysis of the decomposition of both ammonium perchlorate and ammonium nitrate by Cu_2_O, as well as for the accompanying acceleration of combustion processes.
- Copper(II) oxide, CuO, can accelerate the thermal decomposition of AN via electron activation and increasing the phase stability of the oxidising agent, making it a promising catalytic additive in “green” propellant formulations accodring to recent literature reports [35].
- Zinc oxide, ZnO, significantly decreased the AN decomposition activation energy in a binary system; however, the influence of this oxide on the power output (Table 4) and combustion velocity was minor. This may stem from the fact that the decomposition of AN in the presence of ZnO is reported to take place at approx. 130 °C, resulting in earlier evolution of oxygen and its deficiency in the later stages of combustion [33,36]. Consequently, despite strongly promoting AN decomposition, ZnO does not have a comparably favorable effect on SRP combustion to the studied copper oxides.
- Iron(III) oxide, Fe_2_O_3_, in turn has a less notable effect on AN decomposition in binary mixtures than it does on the power output of the SRP. Simultaneously, however, it significantly increases the sensitivity of the SRP to friction (Table 1), largely due to its high hardness (Table 2). The presence of such hard “grit” is known to promote the formation of local ignition points (“hot spots”) under friction [17] and may play a significant if not strictly catalytic role in the SRP samples.

Literature reports [37,38] confirm that the presence of metal oxides in systems containing ammonium perchlorate or ammonium nitrate has a significant effect on the thermal decomposition profile, activation energy, and mechanical sensitivity of the system, which is in line with the trends observed in this work. Unfortunately, the indicated reports provide few clues as to the mechanism by which these effects take place.

To summarise, whereas Cu_2_O and Fe_2_O_3_ exhibit the highest utility as combustion modifiers, their mechanism of action differs significantly. Cu_2_O has a notable catalytic effect, lowering the AN decomposition reaction activation energy and facilitating redox reactions. Conversely, Fe_2_O_3_ increases the propagation rate of the combustion front (i.e., increased linear combustion velocity) and power output of the SRP. In turHn, the case of ZnO shows that the utility of the catalyst cannot be judged solely based on the activation energy—the synchronisation of oxidising agent decomposition and fuel oxidation remains a crucial factor.

It is quite difficult to compare the obtained results with different classes of catalysts because there are no reports in literature on the application of these substances with AN; existing publications are mainly focused on AP-based propellants [5,39]. Nevertheless, the selection of a catalyst should be guided by sufficiently high stability and robustness (both thermal and chemical), low cost, and ease of processing, particularly in large-scale production processes associated with propellant manufacturing.

### 2.4. Identification of Solid Post-Combustion Residues

The rocket propellants formulations were prepared in such a way as to achieve a negative oxygen balance, which is typical for CRP [40,41]. In the context of the research we performed, this was relevant to assess the effect of the oxides used under oxygen-deficient conditions, particularly as solid carbon residues fill the pores on the catalyst’s surface and hinder its catalytic activity.

In light of the above assumptions, we have investigated the solid post-combustion residue using X-ray diffractometry and Raman spectroscopy.

**Figure 6:**
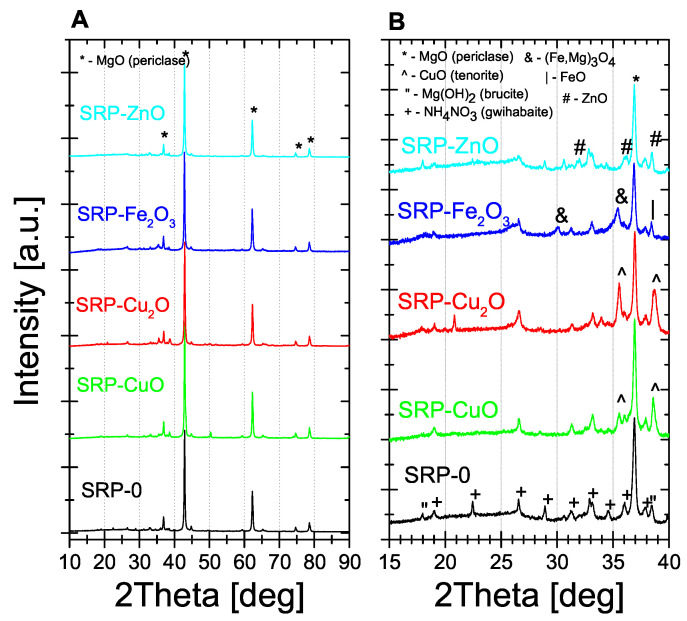
X-ray diffraction patterns of decomposition products in 10–90 deg (**A**) and 15–40 deg (**B**) 2Theta scale ranges.

The XRD patterns of the five post-combustion residues are presented in Figure 6A in the full 2θ scale range. The main MgO periclase (PDF Card No.: 01-071-3631) phase is identified in all products. However, the limited 2Theta scale range (15–40 deg) XRD patterns are shown in Figure 6B for deeper insight into minor phase content. From the inspection of small peaks in this 2Theta range, there is evidence of the presence of unreacted NH_4_NO_3_-type (PDF Card No.: 01-085-0600) phase, especially in the “AN” sample, some CuO tenorite (PDF Card No.: 01-076-7800) phase in “CuO” and “Cu_2_O” samples, ZnO (PDF Card No.: 01-078-4606) phase in the “ZnO” sample, and FeO (PDF Card No.: 01-086-8051) and Fe_3_O_4_-type (PDF Card No.: 01-076-9742) phases in the “Fe_2_O_3_” sample. A tiny amount of the magnesium hydroxide Mg(OH)_2_ brucite phase (PDF Card No.: 01-071-5972) was also found in all the samples. Figure 6B shows a slight rise in the baseline, which may suggest the presence of an amorphous phase, which is probably the incompletely burnt polymer. However, in order to confirm our assumptions we performed analysis with Raman spectroscopy.

**Figure 7:**
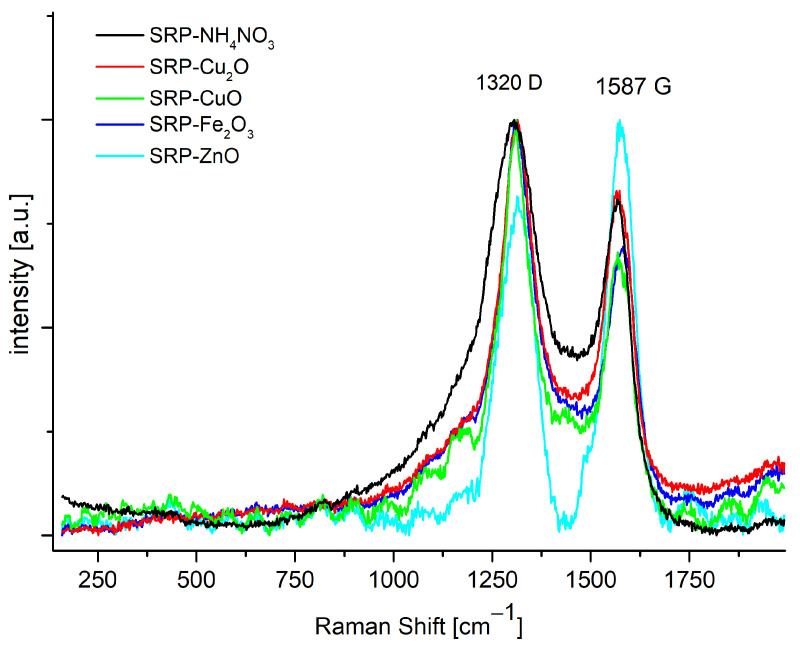
Raman spectra of the investigated post-combustion residues.

Raman spectra of post-combustion residues (Figure 7) show the characteristic signals of amorphous carbon, consisting of two peaks (i.e., the G graphite and disordered D bands), where D is the defect band (around 1330 cm^−1^) and G is the optical phonon of carbon atoms moving in phase opposition around (1580 cm^−1^). This confirms that under the reaction conditions, the cured polymer binder does not burn completely. The absence of signals for inorganic oxides, which were observed by XRD, results from the very high sensitivity of RM spectroscopy to the presence of carbon structures, whose signals by far exceed the magnitude of the signals originating from inorganic species. Consequently, the technique, unlike XRD, allows for the verification of the complete combustion of the cured GAP.

The presence of multiple minor crystalline phases, as seen in XRD patterns, suggests partial transformation or decomposition of the oxide catalysts. However, since the formulations are designed for single-use applications, the influence on catalytic reusability is limited. Nevertheless, such residues may impact the environmental profile of post-combustion solids and should be considered in further assessments.

## 3. Materials and Methods

The exact list of reagents used for the preparation of rocket propellant samples is presented in Table 5.

**Table 5:** Materials used in this work.

Chemical (Code)	Purity Grade	Source	Notes
Ammonium nitrate (AN)	>95%	POCH S.A (Gliwice, Poland)	0.25–0.1 mm ^1^
Iron (III) oxide	>95%	WARCHEM (Zakret, Poland)	83 nm ^2^
Copper (I) oxide	>95%	WARCHEM (Zakret, Poland)	345 nm ^2^
Copper (II) oxide	>95%	WARCHEM (Zakret, Poland)	613 nm ^2^
Zinc oxide	>95%	POCH S.A (Gliwice, Poland)	227 nm ^2^
Glycidyl azide polymer (GAP)			Synthesised as per [42]
Magnesium	>95%	POCH. S.A (Gliwice, Poland)	89 μm ^3^
Methylene diphenyl diisocyanate (MDI)	>95%	Sigma Aldrich (Saint Louis, MO, USA)	Used as a cross-linking agent
Nitroguanidine (NQ)			Synthesised as per [43]
Dibutylin dilaureate	>95%	Sigma Aldrich (Saint Louis, MO, USA)	Used as a catalyst for cross-linking reaction

^1^ Particle size was determined using sieve analysis. ^2^ Particle dimensions were determined using Dynamic Light Scattering (DLS). Ethylene glycol was used as a medium to prepare the oxide suspension. ^3^ Information provided by manufacturer.

### 3.1. Preparation of Rocket Propellant Samples

Each of the samples was prepared with the same procedure. Each of the samples was prepared in a 50 mL glass beaker and mixed by hand using a glass dipstick.

The components of the rocket propellants, taking into account the mass percentage in which they were present in a given sample, are summarised in Table 6.

We applied a metal oxide additive at a level of 2 wt. % based on existing widespread practice [44,45,46]. Further development of the SRP formulations, however, necessitates optimising the catalytic oxide additive content and elucidating the dose–effect relationship of this content and the achieved benchmarks of the SRP formulations.

### 3.2. Determination of Friction Sensitivity

Friction sensitivity was determined on the Peters Friction Apparatus. This determination involves applying a small amount of the test sample to a porcelain stamp and lowering the arm of the apparatus. The porcelain punch is attached to the arm in such a way that when the arm of the apparatus is lowered, it remains in contact with the test material. In addition, a weight of a certain mass is placed on the arm of the apparatus in such a way as to induce a certain pressure force. The movable table on which the plate with the material under test is placed, making a forward–backward movement, causes friction. The test is carried out under varying pressure forces until no sign of reaction is observed when the test is repeated six times.

### 3.3. Determination of Thermal Parameters of Tested Rocket Propellant Samples

In order to trace the energetic reaction that rocket propellants undergo, an analysis was performed using the DTA/TG MOM Q 1500D System Paulik–Paulik–Erdey (MOM, Budapest, Hungary). Samples of 100 mg were used in the measurements performed in the atmosphere of both air (flow rate: 0 mL/min) and argon (flow rate: 40 L/h, purity 5.0). Alumina crucibles were used and the reference material was Al_2_O_3_. Measurements were performed at a heating rate of 5 K/min, in the temperature range from 20 to 1000 °C. For each sample tested, the tests were conducted twice. The first determination was performed in an air atmosphere, while the subsequent determination was performed in an argon atmosphere. Such an arrangement was intended to replicate the conditions of the non-oxygen atmosphere.

### 3.4. Determination of Ignition/Explosion Temperature

Determination of ignition/explosion temperature was made on an Explosion Temperature Tester. The samples were tested in the temperature range of 100–400 °C, with a constant heating rate of 5 °C/min. The test was repeated 5 times and the result is presented as an average.

### 3.5. Determination of Kinetics Parameters

In order to determine the kinetics of high-energy decomposition of AN, differential scanning calorimetry (DSC) was used. The measurements were conducted using a Mettler Toledo DSC 3 instrument, capable of operating in a temperature range of −70 °C to 900 °C. Samples of 3 ± 0.2 mg were tested and heated in a range of 20–450 °C for different heating rates of 3, 5, 7, 10, 15, and 20 K/min.

By determining the peak temperature (i.e., the temperature at which the endothermic peak appears) at a well-defined heating rate, it is possible to determine the activation energy using Kissinger’s method [47]. The applicability of Kissinger’s method to the investigated SRP formulations is justified by the experimental conditions and the thermal behaviour of the propellants. The DSC measurements were carried out at different heating rates, fulfilling the requirement of linear temperature increase assumed in the Kissinger approach. The recorded DSC curves exhibited a single, well-defined main exothermic peak associated with the dominant decomposition process of the propellant formulations. For all heating rates, the position of the peak shifted systematically towards higher temperatures with increasing heating rate, while maintaining a similar peak shape. This behaviour suggests that the degree of conversion at the peak temperature remains comparable. Based on a literature review, it appears that the Kissinger equation is most commonly used to determine the activation energy for SRPs [37,38,47].

### 3.6. Determination of Linear Combustion Velocity

In this study, electrical techniques were applied to evaluate the linear combustion velocity. The system recorded the time intervals corresponding to signal changes at the inputs, which occurred as the advancing flame front successively severed wires embedded in the sample. For the measurements, the propellant samples were placed inside a cellulose tube with dimensions of h = 8 ± 0.2 cm and ϕ=0.6 cm.

### 3.7. X-Ray Diffraction

X-ray diffraction (XRD) with Cu Kα radiation (α = 1.54183 Å) was performed to identify the crystalline phases of decomposition products using a Rigaku MiniFlex 600 (Rigaku Co., Tokyo, Japan) at RT using a one-dimensional detector (Rigaku D/teX Ultra 250) and zero-background sample holder (monocrystalline Si). The X-ray tube was operated at 40 kV and 15 mA. Additional measurement parameters are 2θ range 10–90°, IHS slit = 10 mm, Soller slits = 2.5°, DS slit = 1.25°, scanning step size 0.01°, and exposure time at each point 1.67 s without sample rotation. Phase identification was performed using the Rigaku PDXL2 software, version 2.9.2.0 package and PDF-2 2025 crystallographic database.

### 3.8. Raman Spectroscopy

The Raman spectra were recorded using the InVia Confocal Raman Spectrometer from Renishaw (Gloucestershire, UK) equipped with a DM2500 microscope from Leica (Wetzlar, Germany). The Raman spectra were collected using a laser wavelength of 532 nm, a laser power of 10 mW, and an exposure time of 2 s. An accumulation number of 10 and a grating groove density of 2400 lines mm^–1^ were used with 20× microscope objectives, and the estimated beam size was approximately 7.8 μm.

## 4. Conclusions

The samples containing metal oxides exhibited lower friction sensitivity compared to the reference sample (SRP-0). The increased friction sensitivity reported for SRP-Fe_2_O_3_ may be a result of its high hardness—this can promote the formation of localised hot spots under frictional loading.

As expected, the catalytic activity of the oxides was confirmed by the reduction in the ignition temperature of the SRPs as well as the reduction in E_a_. Since the lowest E_a_ value was calculated for both ZnO and Cu_2_O, a significant influence of these oxides on estimated power output was also expected. Interestingly, the power output of SRP-ZnO is lower than for Cu_2_O. This indicates that although ZnO exhibits favourable catalytic properties (as confirmed by a significant reduction in the activation energy of AN decomposition), its utility as a combustion promoter is limited. The large difference between T_onset_ and T_peak_ reported for SRP-ZnO may be the reason for its limited effectiveness in enhancing the LCV. According to DSC data, energetic decomposition of SRP-ZnO begins at approx. 130 °C, confirming the strong catalytic effect of ZnO on the oxidising agent. Therefore, oxygen is released at an early stage of decomposition, likely resulting in its deficiency during the later stages of SRP decomposition, ultimately leading to a significantly reduced burning rate.

XRD analyses confirmed that solid post-combustion residues consist mostly of magnesium oxides. Additionally, XRD patterns also showed a small residue of unburned propellant components, mainly the oxidising agent and metal oxides. Raman spectra showed, however, that under the test conditions, the polymer did not burn completely. Interestingly, XRD patterns showed only small raise in the baseline, which suggests that even for a negative oxygen balance, the solid carbon content of the combustion products is not significant. As shown in Table A1, the mass residue (read from DTA/TG thermograms), is consistent with stoichiometric calculations from Equation (1).

During this research, the high influence of the metal oxides used on the properties of SRPs was confirmed. That said, the choice of the utilised metal oxide is not straightforward, as even a low activation energy of the AN decomposition reaction in the AN/oxide system does not ensure favourable performance, as seen by the comparison of power outputs of SRP-ZnO and SRP-Cu_2_O. The studies conducted show that copper(I) oxide and iron oxide significantly improve the performance of the propellant formulations, among the tested oxides. Their catalytic activity results in a lowered activation energy and an increased burning rate, thereby leading to an overall enhancement in the estimated power output.

## Figures and Tables

**Table 1:** Friction sensitivity and ignition/explosion temperatures (n = 5) of the SRP samples.

Sample	FS [N]	Ignition/Explosion Temperature [°C]
SRP-0	120	235 ± 1.9
SRP-Fe_2_O_3_	80	234 ± 1.7
SRP-CuO	108	226 ± 1.9
SRP-Cu_2_O	96	230 ± 1.5
SRP-ZnO	84	235 ± 1.6

**Table 2:** Physicochemical properties of the studied oxides.

Oxide	Mohs Scale Hardness	T_melt._ [K]	Lit.
Fe_2_O_3_	6	Decomposition (1735)	[18]
Cu_2_O	3.8	1516.7	[19,20]
CuO	3.8–4.0	Decomposition (1397)	[20,21]
ZnO	4	1972 ± 25	[22,23]

**Table 3:** Performance of studied formulations.

Formulation	Density [g/cm^3^]	LCV [mm/s]	Power Output [W]
SRP-0	1.52	1.10 ± 0.19	36.5
SRP-Fe_2_O_3_	1.59	2.39 ± 0.20	108.4
SRP-CuO	1.66	1.41 ± 0.10	66.3
SRP-Cu_2_O	1.66	2.56 ± 0.20	107.7
SRP-ZnO	1.62	1.68 ± 0.04	55.8

**Table 4:** Determined activation energy with Kissinger’s method.

Sample	Determined $E_{A}$ [kJ/mol]
NH_4_NO_3_	154 ± 12
NH_4_NO_3_ + Fe_2_O_3_	136 ± 14
NH_4_NO_3_ + CuO	134 ± 8
NH_4_NO_3_ + Cu_2_O	122 ± 3
NH_4_NO_3_ + ZnO	123 ± 7

**Table 6:** Constituents of tested rocket propellant samples.

Components wt. %	SRP-0	SRP-Fe_2_O_3_	SRP-CuO	SRP-Cu_2_O	SRP-ZnO
Ammonium nitrate (AN)	54	52	52	52	52
Iron (III) oxide	0	2	0	0	0
Copper (I) oxide	0	0	0	2	0
Copper (II) oxide	0	0	2	0	0
Zinc oxide	0	0	0	0	2
Glycidyl azide polymer (GAP)	25	25	25	25	25
Magnesium (Mg)	16	16	16	16	16
Methylene diphenyl diisocyanate (MDI)	3	3	3	3	3
Nitroguanidine (NQ)	2	2	2	2	2

**Figure A1:**
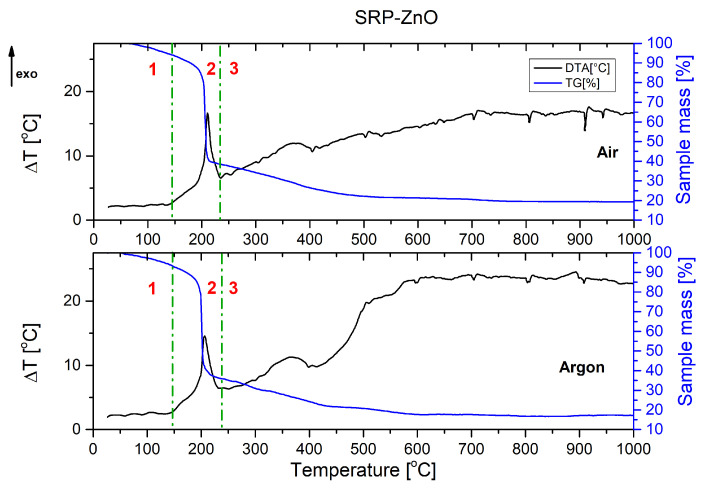
DTA/TG of the sample with zinc(II) oxide (comparison of thermal decomposition occurring in air and in an argon atmosphere). The dashed lines delineate the main combustion event.

**Figure A2:**
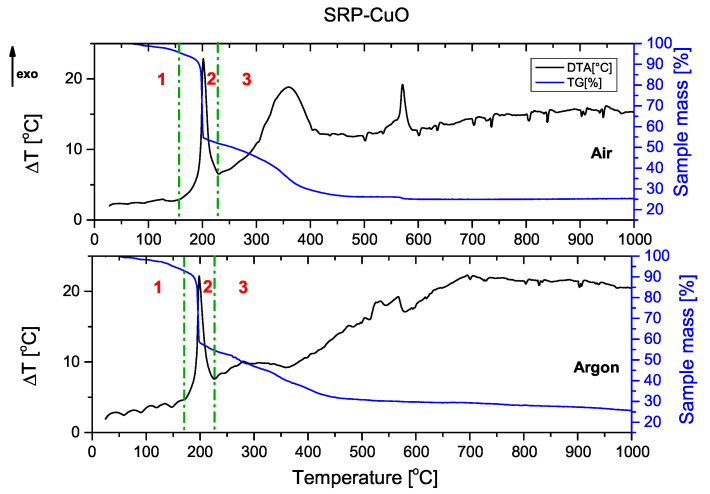
DTA/TG of sample with copper (II) oxide (comparison of thermal decomposition occurring in air and in an argon atmosphere). The dashed lines delineate the main combustion event.

**Figure A3:**
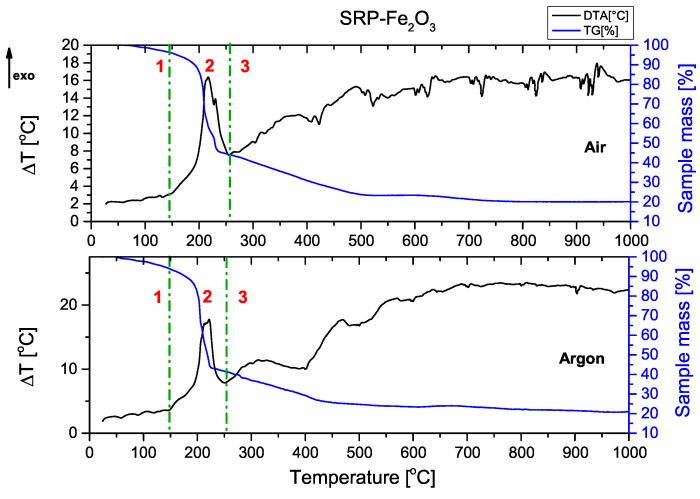
DTA/TG of the sample with iron(III) oxide (comparison of thermal decomposition occurring in air and in an argon atmosphere). The dashed lines delineate the main combustion event.

## Data Availability

Data are contained within the article and Supplementary Materials.

## Supplementary Materials

The following supporting information can be downloaded at: https://www.mdpi.com/article/10.3390/molecules31030436/s1.

## Appendix A

**Table A1:** Solid residue calculations.

Sample (Conditions)	Solid Residue [mg]	Theoretical Mass of MgO ^1^	Difference in Actual and Theoretical Value [mg]
SRP-0 (argon)	22.9	28.0	−5.1
SRP-0 (air)	26.0	28.3	−2.3
SRP-Cu_2_O (argon)	24.4	27.1	−2.6
SRP-Cu_2_O (air)	24.8	27.4	−2.6
SRP-CuO (argon)	26.3	27.3	−1.0
SRP-CuO (air)	27.7	28.9	−1.2
SRP-Fe_2_O_3_ (argon)	22.4	28.3	−5.9
SRP-Fe_2_O_3_ (air)	20.4	26.9	−6.5
SRP-ZnO (argon)	18.8	29.0	−10.2
SRP-ZnO (air)	19.7	27.1	−7.4

^1^ Calculated according to the reaction.

(A1)
$$2Mg+O_{2}\to 2MgO$$

Differences in the masses of the solid residue (actual and theoretical) are due to the measuring cup used. For all DTA/TG measurements, an alumina open cup was used, which led to a portion of the sample being blown out after the measurement.
