# Supplementary material for: Impact of Selected Metal Oxides on the Thermodynamics of Solid Rocket Propellant Combustion
Source: Molecules. 2026 Jan 27;31(3):436. doi: 10.3390/molecules31030436 (PMC12899881; doi:10.3390/molecules31030436)
Supplement: Supplementary file 1 [file molecules-31-00436-s001.zip › molecules-4047933-supplementary.pdf]

Supplementary information for article entitled ,, Impact of selected metal oxides on the thermodynamics of solid rocket propellant combustion”

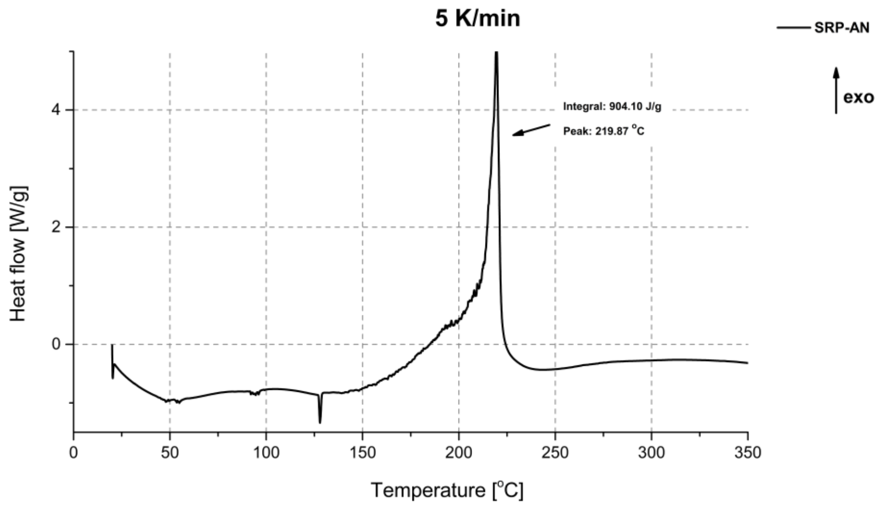

Figure S 1: Thermogram of SRP without addition of metal oxide..

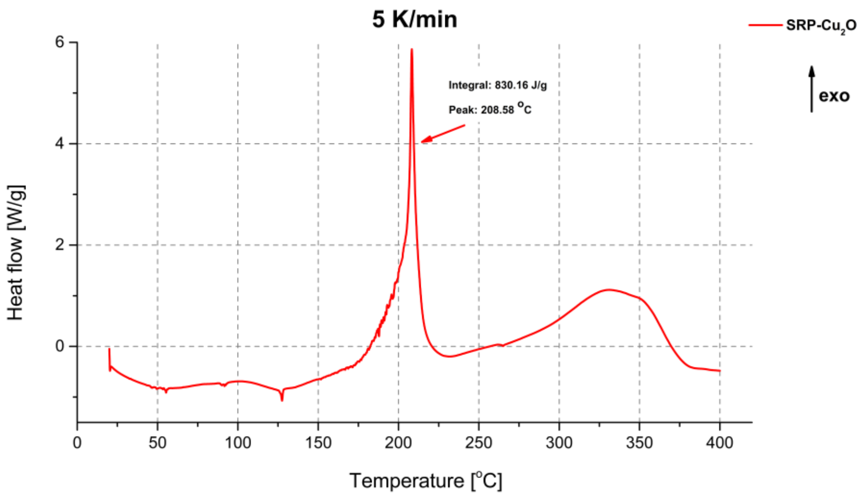

Figure S 2: Thermogram of SRP with addition of Cu_2O.

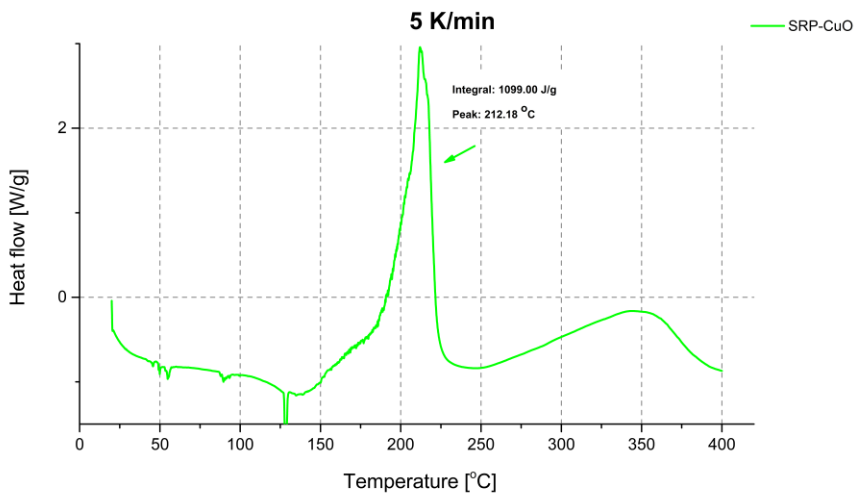

Figure S 3: Thermogram of SRP with addition of CuO.

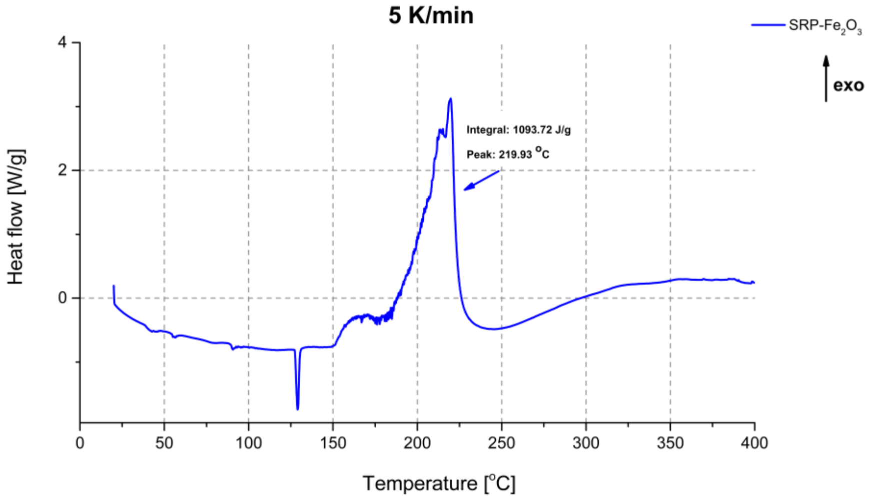

Figure S 4: Thermogram of SRP with addition of Fe_2O_3.

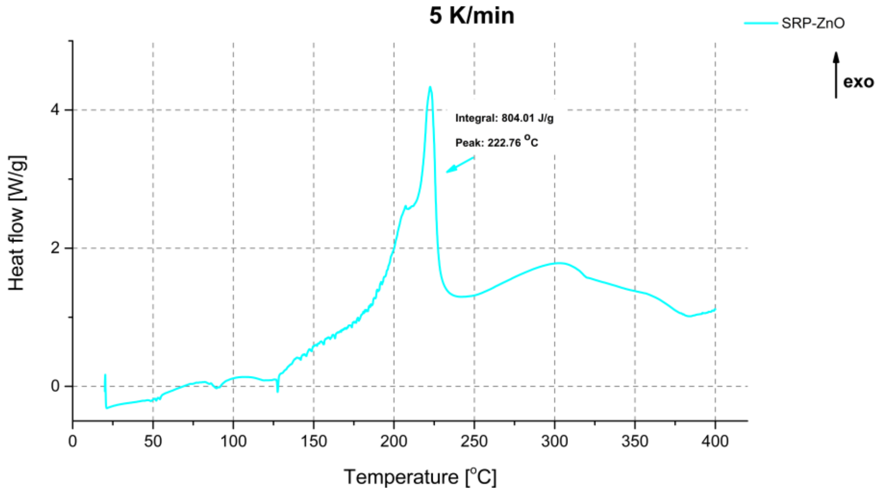

Figure S 5: Thermogram of SRP with addition of ZnO.

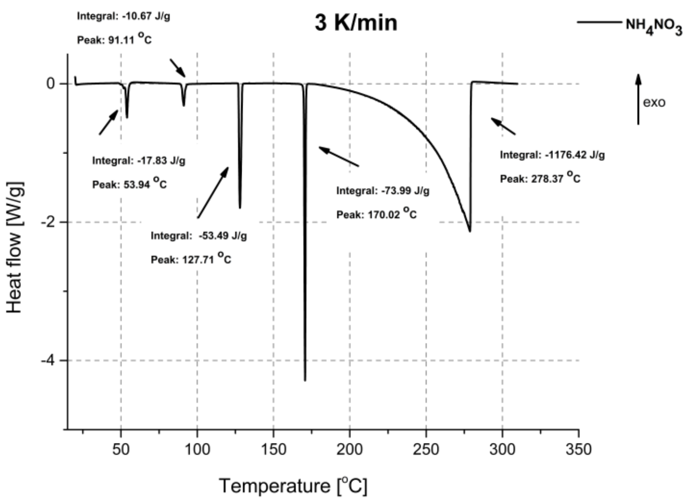

Figure S 6: Thermogram of ammonium nitrate recorded for a heating rate of 3 K/min.

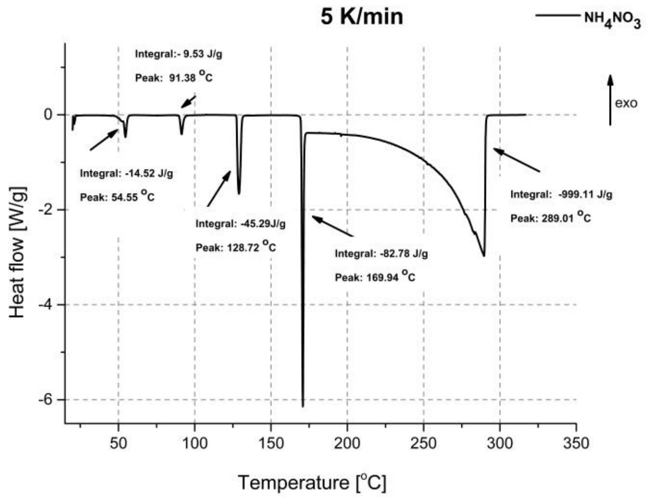

Figure S 7: Thermogram of ammonium nitrate recorded for a heating rate of 5 K/min.

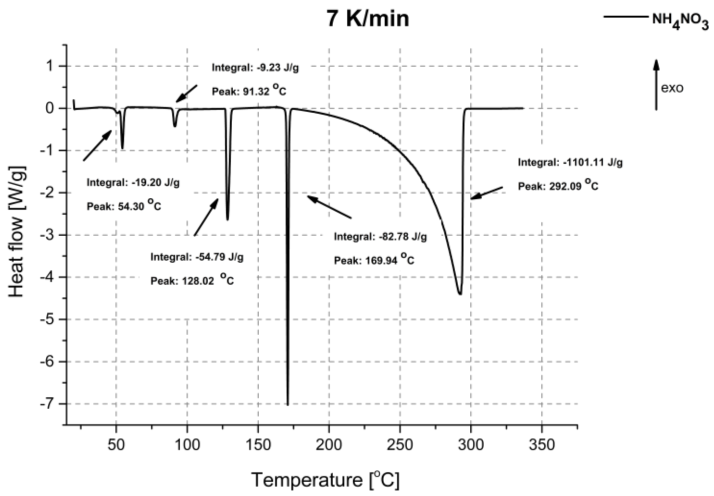

Figure S 8: Thermogram of ammonium nitrate recorded for a heating rate of 7 K/min.

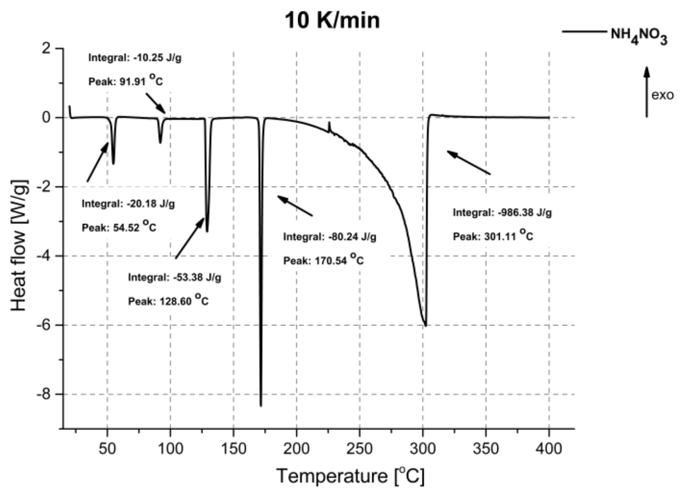

Figure S 9: Thermogram of ammonium nitrate recorded for a heating rate of 10 K/min.

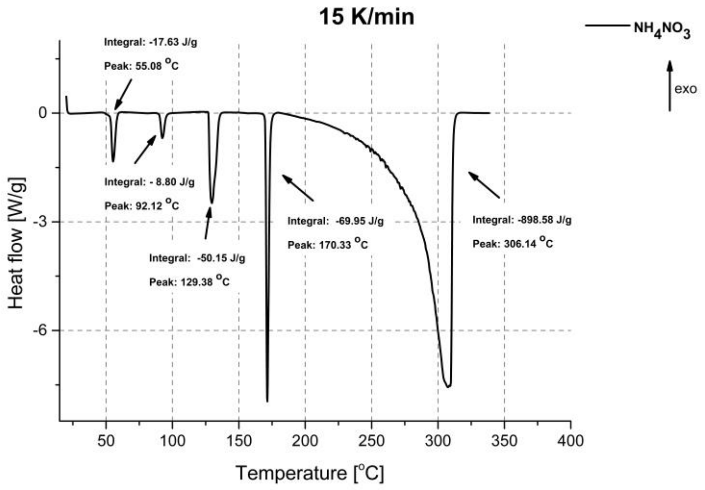

Figure S 10: Thermogram of ammonium nitrate recorded for a heating rate of 15K/min.

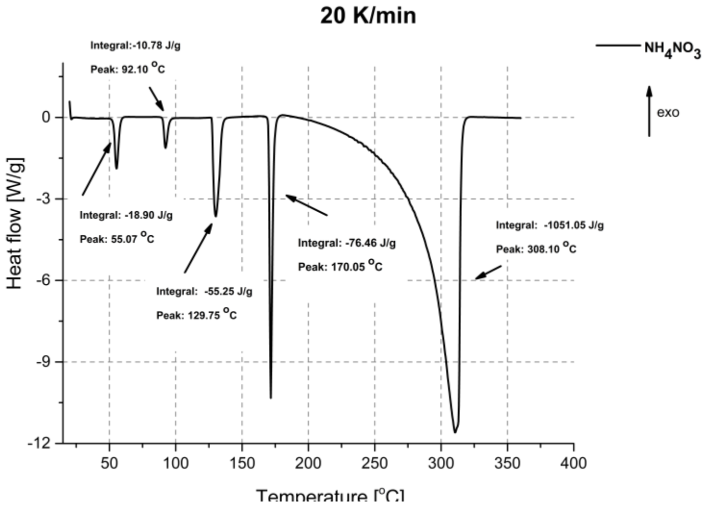

Figure S 11: Thermogram of ammonium nitrate recorded for a heating rate of 20 K/min.

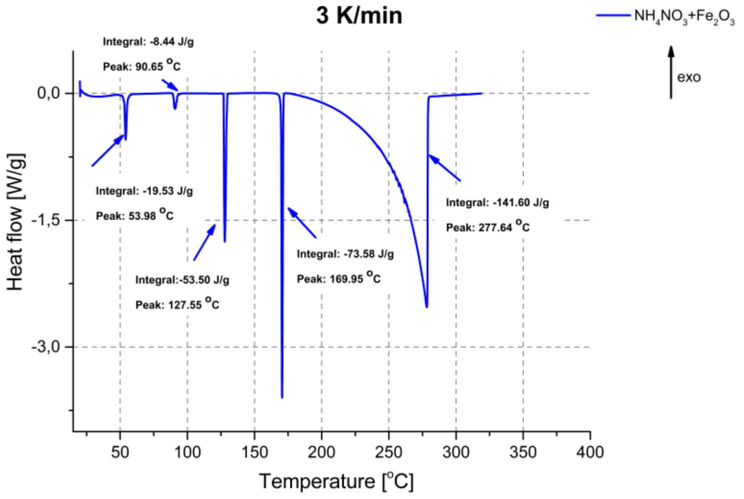

Figure S 12: Thermogram of mixture of iron (III) oxide and ammonium nitrate recorded for a heating rate of 3K/min.

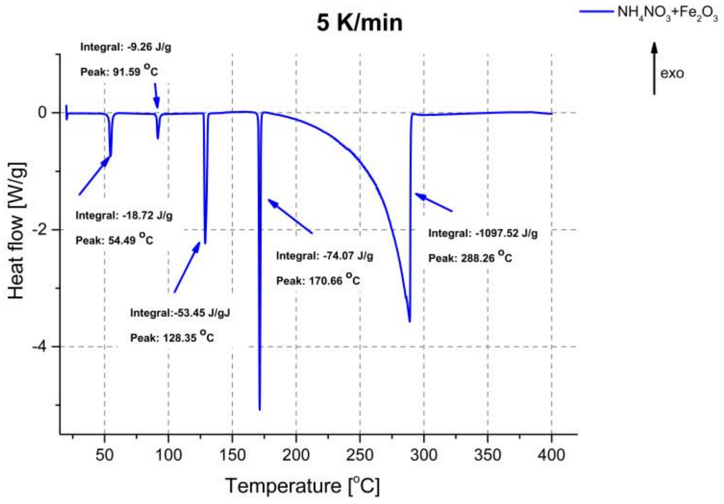

Figure S 13: Thermogram of mixture of iron (III) oxide and ammonium nitrate recorded for a heating rate of 5K/min.

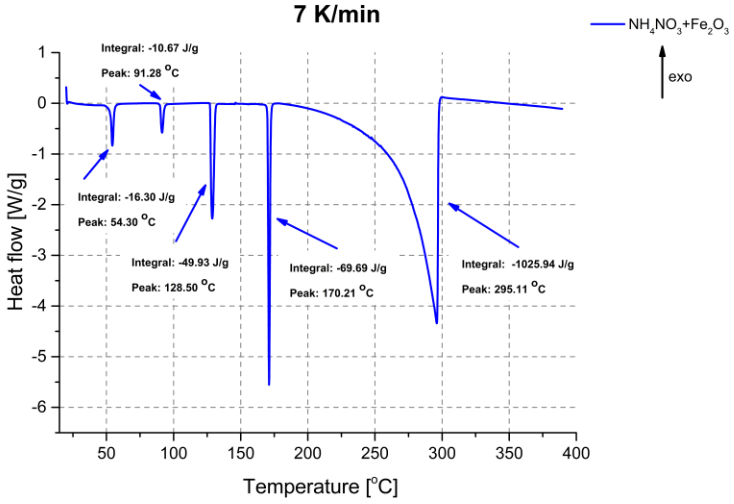

Figure S 14: Thermogram of mixture of iron (III) oxide and ammonium nitrate recorded for a heating rate of 7K/min.

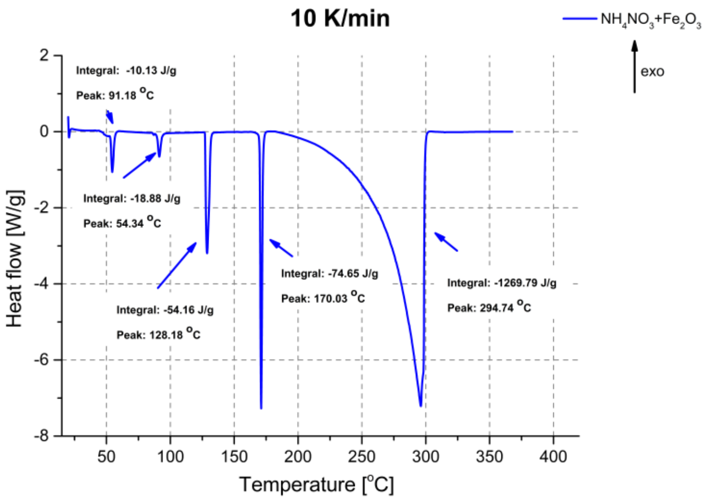

Figure S 15: Thermogram of mixture of iron (III) oxide and ammonium nitrate recorded for a heating rate of 10K/min.

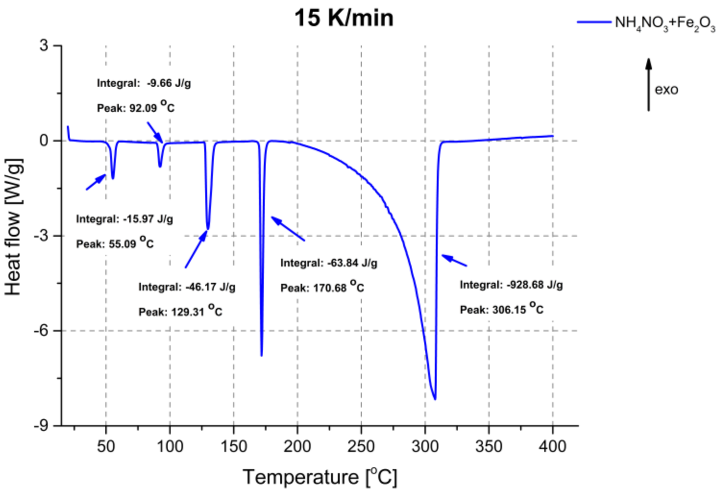

Figure S 16: Thermogram of mixture of iron (III) oxide and ammonium nitrate recorded for a heating rate of 15K/min.

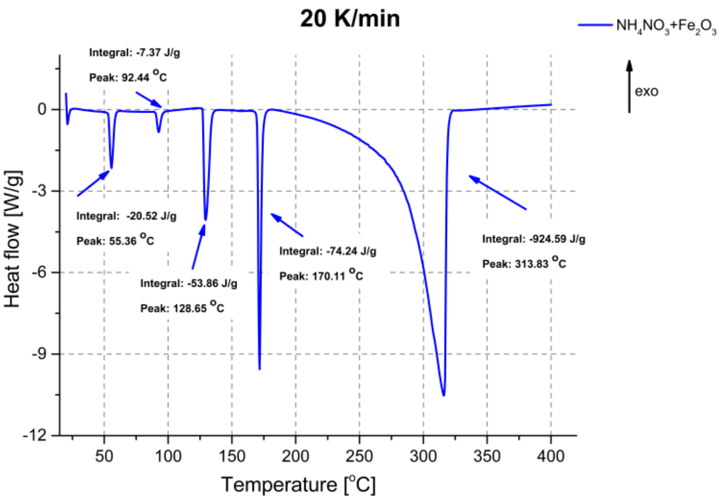

Figure S 17: Thermogram of mixture of iron (III) oxide and ammonium nitrate recorded for a heating rate of 20K/min.

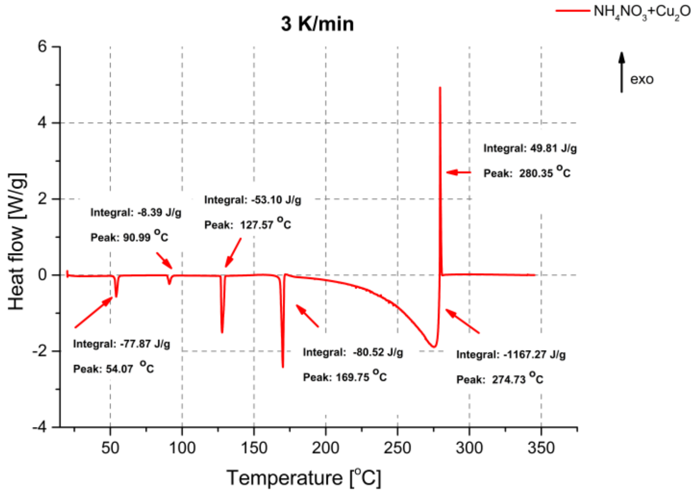

Figure S 18: Thermogram of mixture of copper (I) oxide and ammonium nitrate recorded for a heating rate of 3K/min.

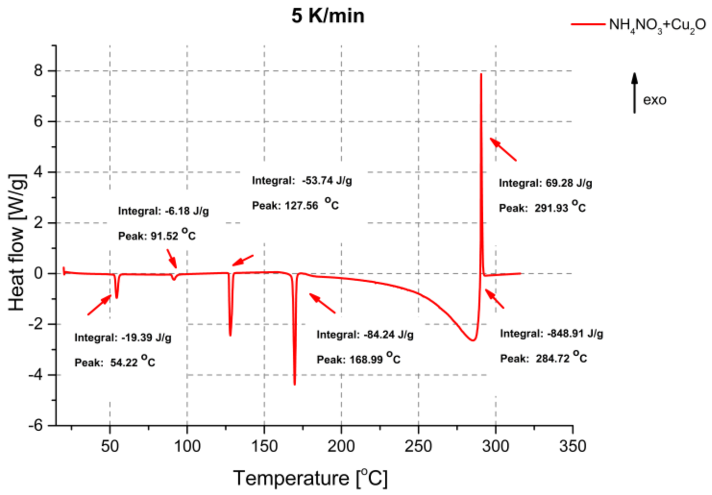

Figure S 19: Thermogram of mixture of copper (I) oxide and ammonium nitrate recorded for a heating rate of 5K/min.

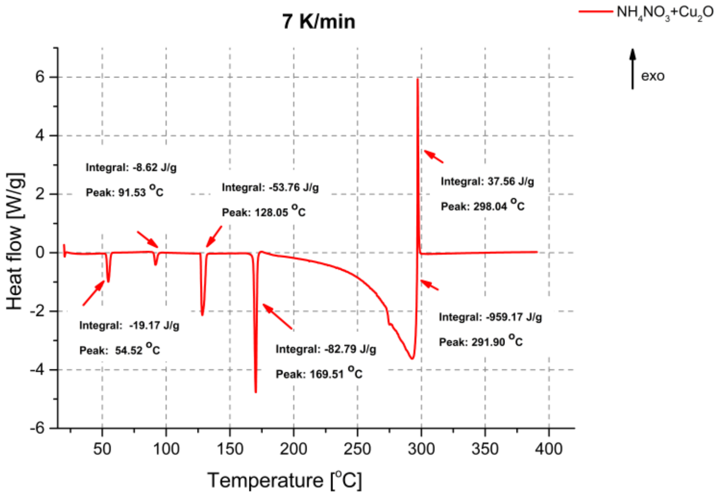

Figure S 20: Thermogram of mixture of copper (I) oxide and ammonium nitrate recorded for a heating rate of 7K/min.

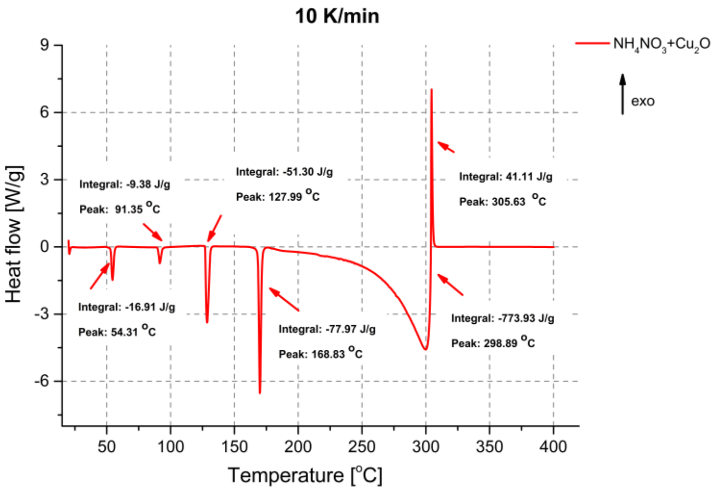

Figure S 21: Thermogram of mixture of copper (I) oxide and ammonium nitrate recorded for a heating rate of 10K/min.

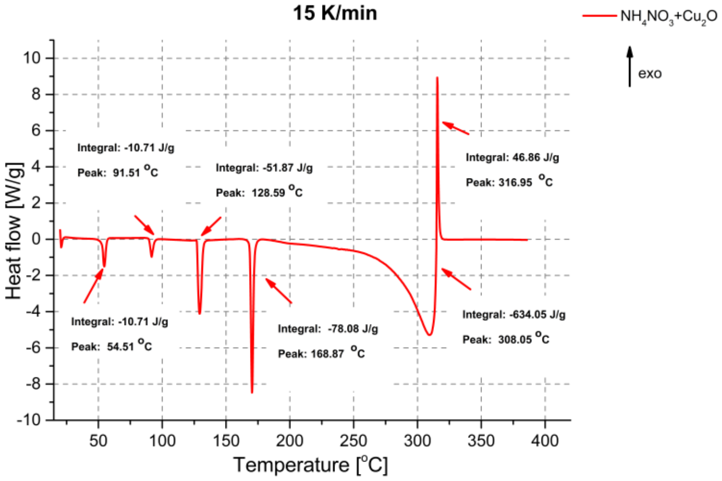

Figure S 22: Thermogram of mixture of copper (I) oxide and ammonium nitrate recorded for a heating rate of 15K/min.

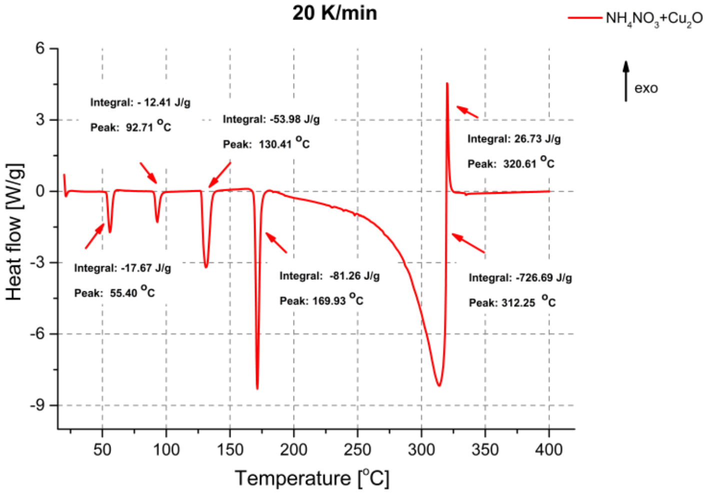

Figure S 23: Thermogram of mixture of copper (I) oxide and ammonium nitrate recorded for a heating rate of 20K/min.

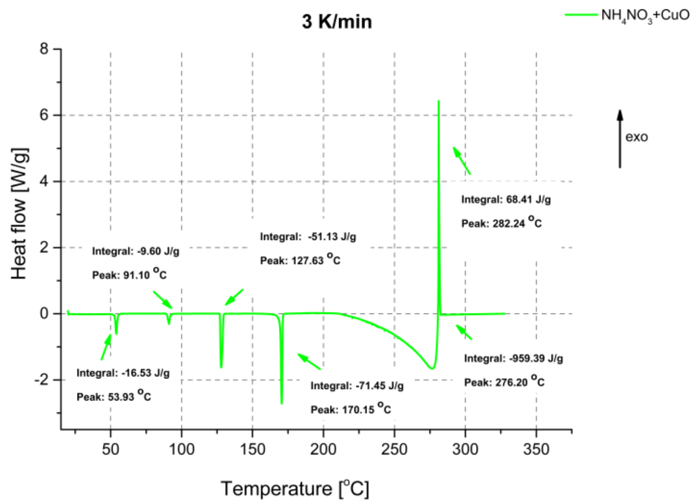

Figure S 24: Thermogram of mixture of copper (II) oxide and ammonium nitrate recorded for a heating rate of 3K/min.

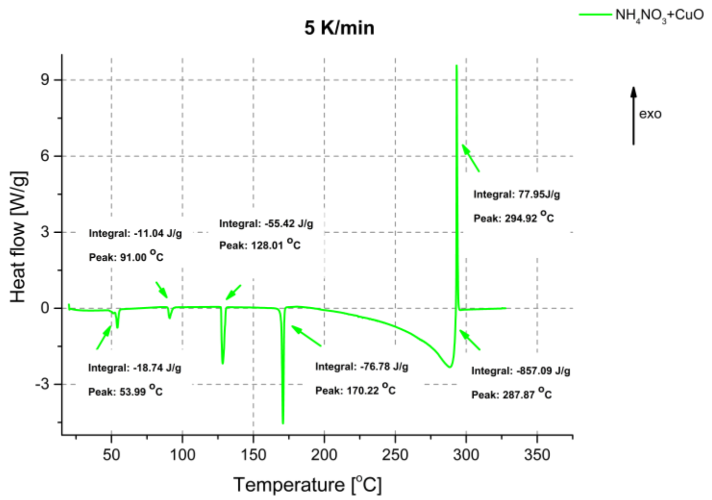

Figure S 25: Thermogram of mixture of copper (II) oxide and ammonium nitrate recorded for a heating rate of 5K/min.

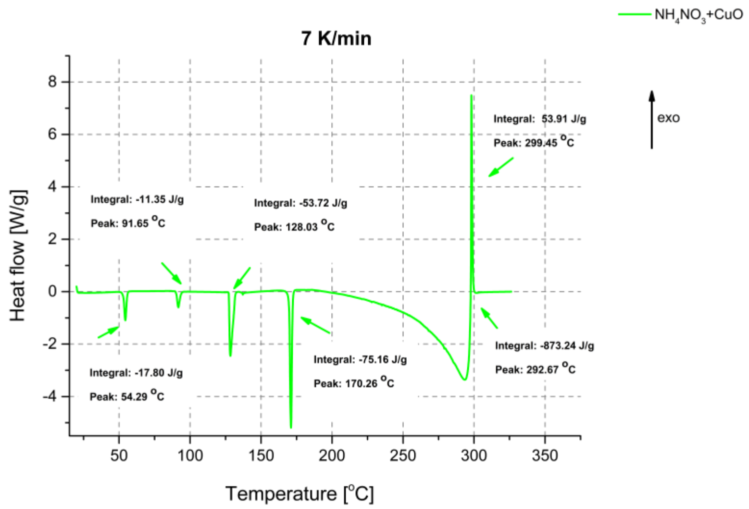

Figure S 26: Thermogram of mixture of copper (II) oxide and ammonium nitrate recorded for a heating rate of 7K/min.

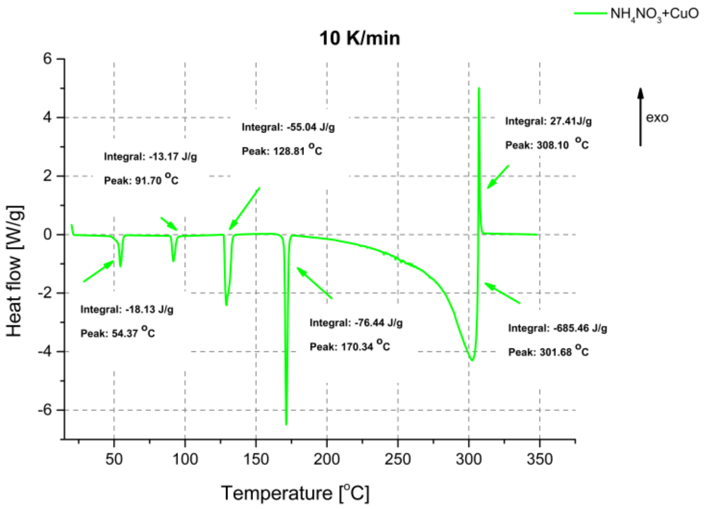

Figure S 27: Thermogram of mixture of copper (II) oxide and ammonium nitrate recorded for a heating rate of 10K/min.

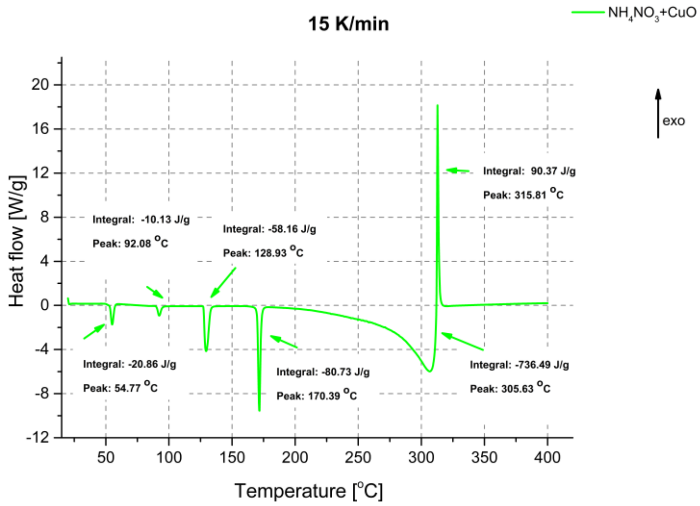

Figure S 28: Thermogram of mixture of copper (II) oxide and ammonium nitrate recorded for a heating rate of 15K/min.

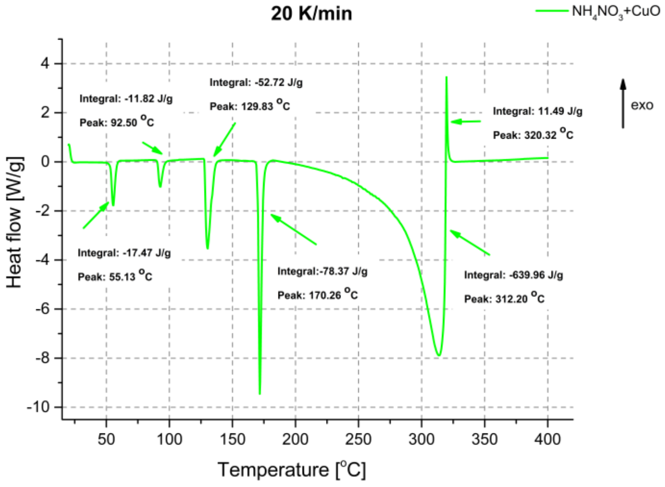

Figure S 29: Thermogram of mixture of copper (II) oxide and ammonium nitrate recorded for a heating rate of 20K/min.

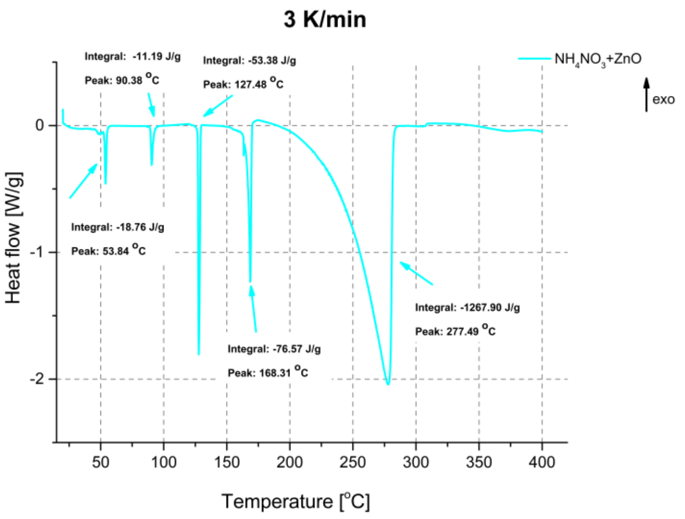

Figure S 30: Thermogram of mixture of zinc oxide and ammonium nitrate recorded for a heating rate of 3K/min.

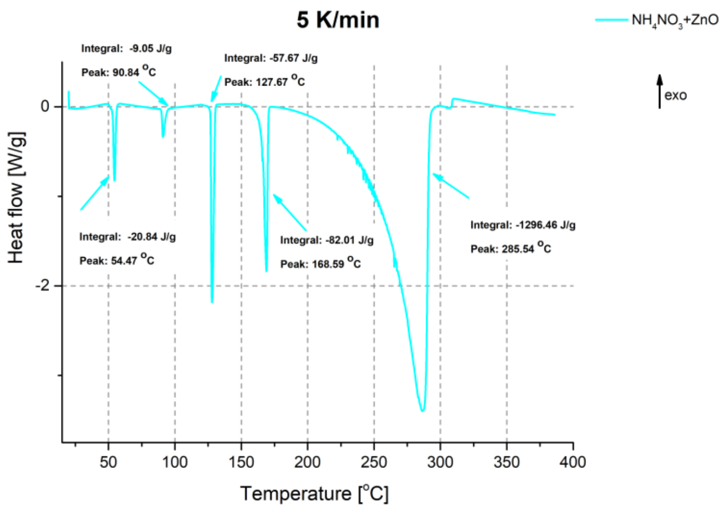

Figure S 31: Thermogram of mixture of zinc oxide and ammonium nitrate recorded for a heating rate of 5K/min.

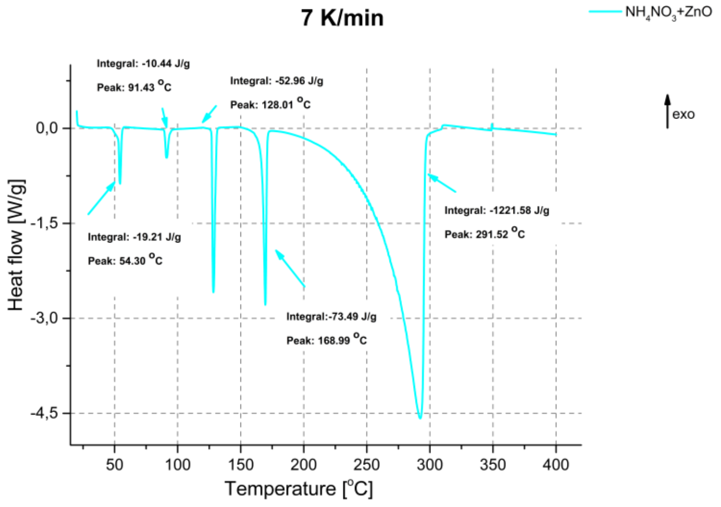

Figure S 32: Thermogram of mixture of zinc oxide and ammonium nitrate recorded for a heating rate of 7K/min.

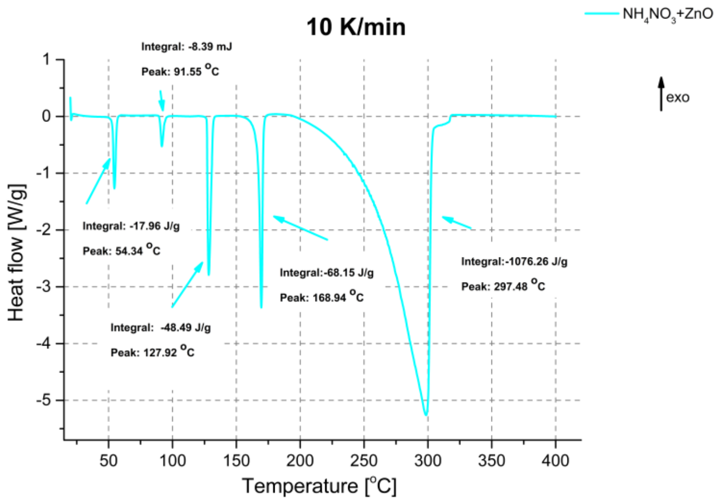

Figure S 33: Thermogram of mixture of zinc oxide and ammonium nitrate recorded for a heating rate of 10K/min.

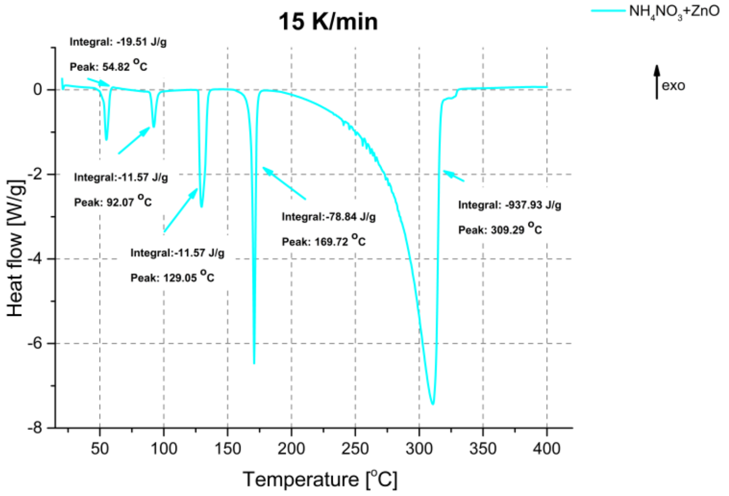

Figure S 34: Thermogram of mixture of zinc oxide and ammonium nitrate recorded for a heating rate of 15K/min.

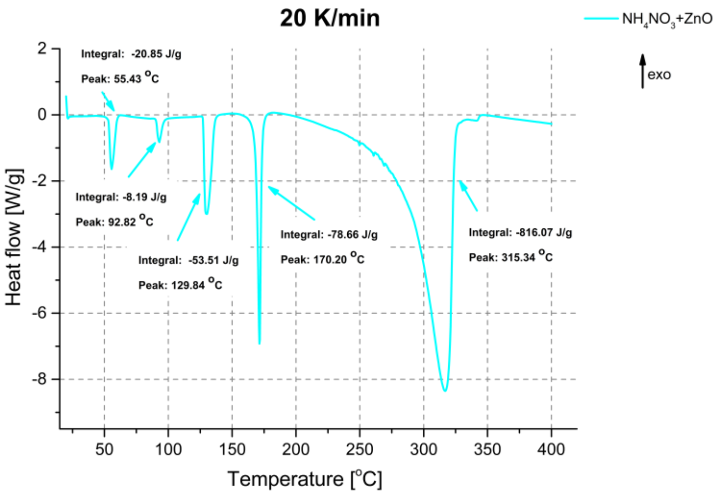

Figure S 35: Thermogram of mixture of zinc oxide and ammonium nitrate recorded for a heating rate of 20K/min.
